# Latent mathematics attitude profiles among eighth-grade students: associations with school belonging and bullying

**DOI:** 10.3389/fpsyg.2026.1938974

**Published:** 2026-09-24

**Authors:** Ramazan Erol

**Affiliations:** Faculty of Education, Afyon Kocatepe University, Afyonkarahisar, Türkiye

**Keywords:** expectancy-value theory, latent profile analysis, mathematics attitudes, peer victimization, school belonging, TIMSS 2023

## Abstract

**Introduction:**

This study examined heterogeneity in students’ mathematics attitudes and the associations of perceived school belonging, bullying victimization, gender, and home educational resources with profile membership.

**Methods:**

TIMSS 2023 Türkiye data included 4,925 eighth-grade students (LPA analytic *N* = 4,820; three-step analytic *N* = 4,739). Latent profile analysis used the three official TIMSS derived scale scores for liking mathematics, confidence in mathematics, and valuing mathematics, with equal indicator variances across profiles and within-profile covariances fixed at zero. Model selection jointly considered fit, profile size, classification quality, substantive distinctiveness, and covariance-specification sensitivity. Associations with profile membership were examined using design-adjusted, classification-error-corrected three-step analyses.

**Results:**

A five-profile solution was retained as a parsimonious descriptive classification: Low Overall / Very Low Confidence, Low Liking-Value / Relatively Higher Confidence, Slightly Below-Average, Positive, and Highly Positive. Perceived school belonging was the most consistent correlate of profile membership, *F*(4, 68) = 47.74, *p* < 0.001, and home educational resources also differentiated profiles, *F*(4, 68) = 15.61, *p* < 0.001. Gender showed a smaller omnibus association, *F*(4, 68) = 2.88, *p* = 0.029, whereas bullying victimization did not differentiate profiles after simultaneous adjustment for the other covariates, *F*(4, 68) = 1.78, *p* = 0.143. When perceived school belonging was omitted in a sensitivity model, bullying victimization did differentiate profiles, *F*(4, 68) = 6.02, *p* < 0.001.

**Discussion:**

Mathematics attitudes varied mainly in overall level, with limited configurational heterogeneity among the two lowest-attitude profiles. The change in the conditional association with bullying after omitting school belonging does not establish mediation or causal ordering.

## 1 Introduction

Mathematics proficiency is associated with later academic and occupational opportunities. Early mathematics knowledge predicts subsequent achievement, including across the primary and secondary school years (Rittle-Johnson et al., 2017). Higher mathematics achievement has also been linked to higher school completion and later human-capital and labor-market outcomes (Levin and Belfield, 2009).

At the broader system level, cognitive skills measured in international assessments are strongly related to individual earnings, income distribution, and economic growth, beyond conventional indicators such as years of schooling (Hanushek and Woessmann, 2008). Mathematics education is therefore relevant both to students’ educational trajectories and to longer-term human capital development.

Proficiency, however, does not depend on cognitive skills alone. Students’ enjoyment of mathematics, confidence in their ability, and perceptions of the subject’s usefulness are theoretically and empirically related to performance, engagement, and educational choices (Wigfield and Eccles, 2000). A systematic review further shows that mathematics attitudes are multidimensional, related to achievement, and often become less positive during secondary education (Wen and Dubé, 2022). Expectancy-Value Theory (EVT; Wigfield and Eccles, 2000) explains these patterns through two central appraisals: expectations of success, reflected in confidence or self-concept, and subjective task value. Task value includes intrinsic value, such as enjoyment, and utility value, or perceived usefulness, as well as attainment value and perceived cost. The Trends in International Mathematics and Science Study (TIMSS) assesses closely related dimensions through scales measuring students’ enjoyment of, confidence in, and valuing of mathematics (Reynolds et al., 2021). These scales provide a theoretically informed basis for examining how mathematics attitudes are organized.

Much of the existing research examines these dimensions separately. Although variable-centered analyses clarify average relations among constructs, they do not show how different attitudes coexist within the same student. A student may regard mathematics as necessary for a high-stakes examination while having little confidence in their ability to succeed. That configuration is difficult to detect when value and confidence are modeled only as separate variables (Berger et al., 2020). Latent Profile Analysis (LPA) provides a person-centered alternative by identifying subgroups of students with similar configurations of attitudes (Berger et al., 2020). Research using TIMSS and other large-scale assessment data has identified distinct mathematics attitude profiles and shown that such profiles differ in achievement or related student characteristics (Berger et al., 2020; Chon and Kim, 2019).

A person-centered analysis may be especially informative in centralized education systems where examination demands can shape students’ perceptions of the importance of mathematics. Türkiye is one such context. Eighth-grade students prepare for the High School Placement Exam (LGS), the centralized transition system used for secondary-school placement; changes in Türkiye’s transition system have been associated with mathematics achievement (Gür et al., 2021). Rather than presuming that high-stakes testing produces a particular motivational profile, this context motivates an empirical examination of whether liking, confidence, and valuing align or diverge within students.

The Turkish context is also relevant because high-stakes examination policies have been linked to the creation and reproduction of educational inequalities (Caner and Bayhan, 2020). Analyses of the 2020 LGS population further show substantial achievement differences associated with student and school background factors (Suna et al., 2021).

Mathematics has substantial weight in the LGS, which may heighten students’ perceptions of its importance for future educational opportunities. At the same time, the extent to which perceived value aligns with confidence and liking is an empirical question. Prior work shows that associations between mathematics motivation and performance can differ under high- versus low-stakes testing (Simzar et al., 2015), and that high mathematics achievement can coexist with less positive affect in a high-performing East Asian context (Pang and Seah, 2021). Socioeconomic and school-context variables are also associated with mathematics motivational beliefs and achievement in Türkiye (Niehues et al., 2020; Suna et al., 2021). These contextual features provide a rationale for examining mathematics attitudes with a person-centered model without assuming in advance which configurations should emerge.

The correlates examined in this study reflect social and educational conditions relevant to eighth-grade students. Perceived school belonging is associated with motivation, self-efficacy, engagement, and achievement during secondary education (Korpershoek et al., 2020). Bullying victimization has been linked to poorer psychosocial well-being through school belonging and self-efficacy (Seon and Smith-Adcock, 2021), and meta-analytic evidence links victimization with lower academic outcomes through cognitive-motivational processes (Samara et al., 2021). Home educational resources reflect differences in students’ learning environments, and gender may differentiate competence-related beliefs even when achievement differences are limited (Rodríguez et al., 2020). The present study treats these variables as concurrent correlates of latent profile membership rather than as causal determinants.

Expectancy-Value Theory (EVT; Wigfield and Eccles, 2000) provides the most direct theoretical mapping to the three mathematics attitude indicators, but the TIMSS scales are not treated as direct operationalizations of EVT constructs. Students Confident in Mathematics is interpreted as expectancy-related, Students Value Mathematics as value-related, and Students Like Learning Mathematics as positive affect/liking that overlaps conceptually with intrinsic value without being identical to it. Self-Determination Theory (SDT; Ryan and Deci, 2000) is used only as an interpretive lens for the social context. The TIMSS school-belonging scale captures students’ individual perceptions of acceptance and connection; it is not a direct measure of SDT relatedness-need satisfaction, autonomy, or self-determined motivation. Bullying victimization, home educational resources, and gender are treated as contextual correlates rather than as direct EVT or SDT measures.

Family background and gender are also established correlates of mathematics learning and motivation. Home resources and mathematics attitudes have both been found to predict mathematics achievement in TIMSS analyses across South Korea, Türkiye, and the United States (Geesa et al., 2019). Gender differences are less uniform: girls may perform comparably to boys while reporting lower mathematics confidence (Mejía-Rodríguez et al., 2021; Rodríguez et al., 2020). Examining these variables together with students’ own perceptions of school belonging and bullying experiences permits a more complete assessment of how student characteristics are associated with mathematics attitude profiles.

### 1.1 Significance of the study

Despite evidence linking school belonging, bullying, gender, and home resources to mathematics-related outcomes, limited research has considered these factors together as correlates of distinct attitude profiles. The contribution of the present study is not to establish that mathematics attitudes are multidimensional, but to examine how the established dimensions of liking, confidence, and valuing combine within students and how those configurations relate to individual social-relational experiences and background characteristics. This distinction matters because students with similarly low overall attitudes may differ in whether confidence, liking, or value is especially low. The Turkish TIMSS 2023 sample provides an opportunity to examine these questions at the transition to secondary education in a centralized, high-stakes context. The study addressed two research questions:

1. Which latent profiles of mathematics attitudes can be identified among Turkish eighth-grade students based on the three official TIMSS scale scores for liking, confidence, and valuing mathematics?
2. To what extent are individual perceived school belonging, bullying victimization, gender, and home educational resources associated with membership in these profiles?

By combining a person-centered analysis with student-reported school experiences and background characteristics, the study examines how social and educational conditions are associated with different configurations of mathematics attitudes. Its practical contribution is to formulate profile-informed questions about differentiated support; whether such support improves achievement, persistence, or well-being requires separate investigation.

## 2 Literature review

### 2.1 Mathematics attitudes as a multidimensional construct

Attitudes toward mathematics cannot be reduced to a single positive or negative disposition. Current research treats them as multidimensional, with affective, cognitive, and sometimes behavioral components that jointly influence students’ engagement with mathematics (Wen and Dubé, 2022). Affective components include emotional responses such as enjoyment and anxiety, whereas cognitive components include perceived competence and beliefs about the importance or usefulness of mathematics. These dimensions also show different associations with achievement. Confidence and value tend to be positively associated with mathematics outcomes, while anxiety and other negative affective responses tend to be negatively associated (Wen and Dubé, 2022).

Measurement studies support this multidimensional view. Tapia and Marsh (2004) identified distinct dimensions including self-confidence, value, motivation, and enjoyment, showing that mathematics attitudes comprise related but separable dimensions. Di Martino and Zan (2010) reached a similar conclusion through students’ narratives. Their work showed that students’ relationships with mathematics vary in emotional disposition, perceived competence, and their view of mathematics as a discipline. Negative attitudes can therefore take different forms rather than constituting a single undifferentiated response.

### 2.2 Liking mathematics, confidence, and valuing as distinct but related dimensions

The present study focuses on liking mathematics, confidence in mathematics, and valuing mathematics. Liking refers to enjoyment, interest, and positive emotional engagement. Confidence concerns students’ beliefs about their competence and expected success. Valuing refers to the importance, usefulness, or personal worth that students assign to mathematics. The dimensions are related, but each describes a different aspect of students’ attitudes.

Empirical findings support these distinctions. Pinxten et al. (2014) showed that enjoyment and competence beliefs were separable and had different associations with achievement and effort. Students may enjoy mathematics without feeling highly competent, or feel competent without strongly liking it. Longitudinal person-centered research likewise indicates that enjoyment, confidence, and value can form different configurations and follow different developmental patterns (Feng et al., 2024). Putwain et al. (2021) found that higher control and value predicted mathematics achievement directly and indirectly through higher enjoyment and lower anxiety. These findings support examining the three dimensions separately while also considering how they combine within students.

### 2.3 Theoretical grounding for confidence and valuing

Expectancy-value perspectives provide the main theoretical basis for examining confidence and value together. Confidence in mathematics is conceptually close to expectancy for success, whereas valuing reflects judgments about the subject’s usefulness, importance, and worth. EVT proposes that expectancies and task values are related to achievement, engagement, persistence, and educational choices (Wigfield and Eccles, 2000; Eccles and Wigfield, 2020). Perceiving mathematics as useful may support effort, while expecting success may help students engage with difficult tasks and persist when they encounter setbacks.

Research on self-beliefs further supports confidence as a distinct dimension. Mathematics self-efficacy predicts performance in mathematical problem solving (Pajares and Miller, 1994) and contributes to performance beyond general ability (Pajares and Kranzler, 1995). Evidence from TIMSS and PISA also identifies confidence-related self-beliefs as strong noncognitive correlates of mathematics achievement (Stankov and Lee, 2017). Together, expectancy-value and self-belief perspectives justify examining confidence and value jointly without treating them as interchangeable. Liking mathematics remains an affective dimension that may or may not align with competence beliefs or perceived utility.

### 2.4 School belonging and bullying as social-relational correlates

School belonging is a social and relational resource for academic functioning. It refers to students’ sense that they are accepted, valued, and included in the school community. Meta-analytic evidence links stronger belonging with motivation, self-beliefs, engagement, and achievement during secondary education (Korpershoek et al., 2020). This relationship may be especially relevant in mathematics, where difficulty and uncertainty can prompt negative self-evaluations. A stronger connection to school may help students remain engaged and maintain more positive attitudes when they encounter these challenges.

Bullying victimization may undermine both school belonging and academic motivation. Samara et al. (2021) identified cognitive and motivational processes, including self-beliefs, engagement, and school connectedness, in the association between victimization and academic outcomes. Other studies have tested indirect pathways involving belonging or teacher-student relationships (Li et al., 2020; Ren et al., 2025). These findings provide a rationale for examining bullying and belonging in the same model; however, the present cross-sectional analysis estimates concurrent conditional associations and does not test mediation.

### 2.5 Home educational resources and gender as contextual correlates

Home educational resources are one component of the wider learning environment. They include material supports such as books, technology, and study space, as well as family conditions that can support engagement and educational aspirations. Evidence from large-scale assessments consistently links socioeconomic background and home resources with educational achievement (Broer et al., 2019). In Türkiye, socioeconomic factors are associated with both mathematics motivation and achievement (Niehues et al., 2020). Home educational resources may therefore help distinguish students with different mathematics attitude profiles.

Gender is another relevant correlate of mathematics motivation. Girls often report lower confidence than boys even when differences in mathematics achievement are small or inconsistent. Rodríguez et al. (2020) found that gender differences may be more apparent in competence beliefs than in performance. Person-centered studies also show that girls and boys can be distributed differently across attitudinal or motivational profiles. Feng et al. (2024), for example, reported gender variation in mathematics attitude profiles over time. Gender may therefore relate to particular combinations of liking, confidence, and value rather than to a uniformly higher or lower level of mathematics attitudes.

### 2.6 Person-centered research on mathematics attitude and motivation profiles

Most studies of mathematics attitudes use variable-centered models to estimate relations among constructs across the sample. These models are informative, but they can conceal differences in how attitudes are combined within individual students. Person-centered methods such as LPA identify subgroups whose members show similar configurations. This distinction matters because liking, confidence, and valuing do not necessarily move together.

Previous person-centered research supports this premise. Gaspard et al. (2019) identified expectancy-value profiles that differed in achievement and educational choices. Lazarides et al. (2019) showed that motivational profiles can change over time and that classroom experiences are associated with transitions between them. Fong et al. (2021) also linked expectancy-value profiles with academic and STEM-related outcomes. More directly, Feng et al. (2024) found that confidence, value, and enjoyment in mathematics formed distinct profiles that varied over time and by gender. Students thus differ not only in the overall positivity of their attitudes but also in the way positive and negative beliefs and feelings are combined.

### 2.7 Gap in the literature and rationale for the present study

Several questions remain despite growing recognition that mathematics attitudes are multidimensional and heterogeneous. Liking, confidence, and value are still commonly examined separately or in variable-centered models, leaving less evidence about the profiles they form within students. School belonging, bullying, home resources, and gender have each been linked to mathematics-related outcomes, but they are seldom considered together as correlates of profile membership. Recent person-centered evidence is also limited for Turkish eighth-grade students participating in an international large-scale assessment.

This study addressed these questions with TIMSS 2023 data from Turkish eighth-grade students. The official TIMSS derived scores for liking mathematics, confidence in mathematics, and valuing mathematics were used as profile indicators. Students’ perceived school belonging, bullying victimization, home educational resources, and gender were then examined as individual-level correlates of profile membership.

## 3 Materials and methods

This quantitative study used a cross-sectional design and data from TIMSS 2023, administered by the International Association for the Evaluation of Educational Achievement (IEA). The following sections describe the sample, measures, and analytical procedures.

### 3.1 Sample and data collection

The analysis used the Turkish national sample from TIMSS 2023. The sample comprised 4,925 eighth-grade students (ISCED Level 2) attending 141 middle schools. Among students with valid gender responses (*N* = 4,845), 49.4% were girls (*n* = 2,393) and 50.6% were boys (n = 2,452). The official TIMSS age variable (BSDAGE) verified the unusual but valid observed range of 10.00 to 17.33 years. The unweighted age distribution was M = 13.87, SD = 0.42; the HOUWGT-weighted distribution was M = 13.86, SD = 0.43. Only nine students (0.18%) were outside the 12-16-year range. Age was not used as a profile indicator or covariate. The three-step analytic sample was restricted to students with valid boy/girl coding and complete data on all four covariates (N = 4,739), of whom 2,351 were girls (49.6%) and 2,388 were boys (50.4%), a composition that closely matches the full national sample.

### 3.2 Variables and measures

Latent profile indicators. Three official TIMSS 2023 derived scale scores were used as continuous profile indicators: Students Like Learning Mathematics (BSBGSLM; SLM), Students Confident in Mathematics (BSBGSCM; SCM), and Students Value Mathematics (BSBGSVM; SVM). The revised analysis uses these scale scores directly rather than the 26 individual questionnaire items.

Students Like Learning Mathematics (SLM). BSBGSLM reflects students’ enjoyment of and positive affect toward learning mathematics. Higher official scale scores indicate greater liking of mathematics. The TIMSS scale construction and international scaling procedures are described by Yin and Reynolds (2024).

Students Confident in Mathematics (SCM). BSBGSCM reflects students’ perceived competence and confidence in mathematics. Higher official scale scores indicate greater confidence. The scale is conceptually related to expectancy for success but is reported here using the TIMSS construct name (Yin and Reynolds, 2024).

Students Value Mathematics (SVM). BSBGSVM reflects students’ perceptions of the importance and usefulness of mathematics. Higher official scale scores indicate stronger valuing. The scale is interpreted as a value-related construct and is not relabeled as an instrumental motivational orientation (Yin and Reynolds, 2024).

Students’ Sense of School Belonging. The official TIMSS derived score BSBGSSB was used to represent students’ individual perceptions of acceptance, connection, and belonging at school. Higher scores indicate a stronger perceived sense of school belonging. This variable is treated as an individual-level perception, not as a school-level climate measure (Yin and Reynolds, 2024).

Student Bullying. The official TIMSS derived scale BSBGSB was used. On the official scale, higher scores indicate less frequent bullying victimization. For the three-step regression, BSBGSB was standardized and its sign reversed so that higher analytic scores represented more frequent victimization; odds ratios therefore correspond to a one-standard-deviation increase in bullying victimization. This direction was verified against the TIMSS data labels and scale scores.

Home Educational Resources. The official TIMSS derived score BSBGHER was used as the home-resource covariate. It summarizes educational resources and family-background information in the home context. The official derived scale score was used directly; no researcher-created raw composite or separate internal-consistency estimate was used in the revised analysis (Yin and Reynolds, 2024).

Gender. The official TIMSS gender item (BSBG01) was used as a demographic covariate. Valid responses were coded 0 = girl and 1 = boy for the revised analysis.

### 3.3 Analytical strategy

To address RQ1, weighted latent profile models were estimated from the three official TIMSS scale scores (SLM, SCM, and SVM). Students with at least one observed profile indicator were retained in the observed-data likelihood. Of the full sample, 105 students were missing all three indicators, yielding an LPA analytic sample of N = 4,820. Missingness for SLM, SCM, and SVM was 2.58, 3.39, and 3.09%, respectively.

All final analyses were conducted in Python 3.12. The authoritative script records the Python and package versions, settings, random seeds, input-data SHA-256 hash, and code SHA-256 hash in a machine-readable run manifest and is also supplied through a self-contained Google Colab runner. The weighted equal-variance Gaussian mixture model was implemented with an observed-data expectation–maximization algorithm so that the same measurement model could be re-estimated and class labels aligned across all 142 Full JK2 replicates. Indicator means were freely estimated across profiles, each indicator variance was constrained to equality across profiles, and all within-profile indicator covariances were fixed at zero. Two hundred random starts were used for each one- through eight-profile enumeration model, and the retained five-profile model was re-estimated with 1,000 random starts. Table 1 reports the absolute difference between the best and second-best log-likelihood values across starts. Algorithm checks covered missing-data likelihood equivalence, analytic gradients, classification-error correction under perfect classification, the JK2 variance formula, and class-label alignment. Figures 1, 2 and Supplementary Figure S1 were rendered programmatically from the final machine-readable outputs using Matplotlib 3.10.8.

**Table 1:** Model fit indices for the one- through eight-profile equal-variance latent profile models (LPA N = 4,820).

**k**	**Par**.	**LogLik**	**AIC**	**BIC**	**aBIC**	**Entropy**	**Min. modal wt. %**	**Delta LL (best-2nd)**
1	6	−31201.54	62415.09	62453.97	62434.90	-	100.00	<0.00001
2	10	−29527.15	59074.30	59139.10	59107.33	0.706	48.46	0.00022
3	14	−28815.26	57658.51	57749.24	57704.75	0.771	20.50	0.00003
4	18	−28578.21	57192.42	57309.07	57251.87	0.769	13.42	0.00005
5	22	−28431.18	56906.37	57048.94	56979.03	0.787	5.64	0.00004
6	26	−28311.06	56674.11	56842.61	56759.99	0.753	4.39	0.00138
7	30	−28224.15	56508.31	56702.72	56607.39	0.776	0.68	0.00151
8	34	−28147.14	56362.28	56582.62	56474.58	0.788	0.70	0.00003

k = number of profiles; AIC, Akaike Information Criterion; BIC, Bayesian Information Criterion; aBIC, sample-size-adjusted BIC; ΔLL(best-2nd), absolute difference between the best and second-best log-likelihood values among the enumeration starts. Entropy is not defined for the one-profile model. Information criteria decreased monotonically through eight profiles without a clear elbow; the five-profile solution was retained primarily after considering profile size, classification quality, parsimony, and substantive distinctiveness. The retained five-profile model was re-estimated with 1,000 random starts. A binary count of ‘replicated’ starts is not used as a selection criterion because it depends on the numerical tolerance.

**Figure 1:**
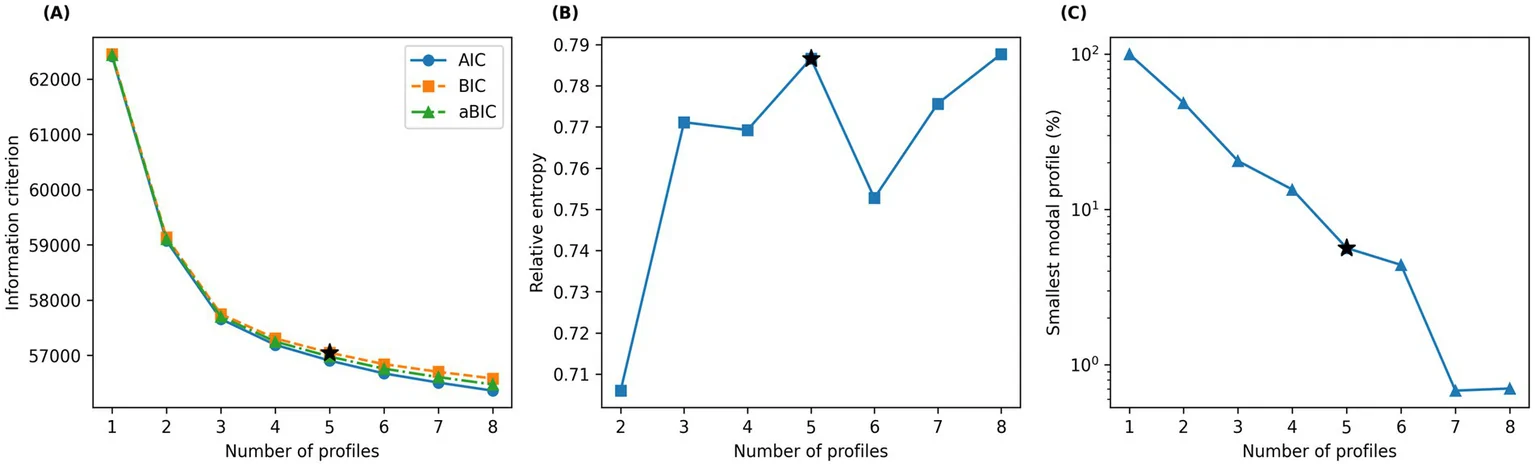
Model comparison for the one- through eight-profile equal-variance latent profile models. Panel **(A)** shows the information criteria, which decline monotonically and provide no minimum within eight profiles. Panel **(B)** shows relative entropy. Panel **(C)** shows the modal weighted prevalence of the smallest profile on a logarithmic scale. The black star identifies the retained five-profile descriptive classification; selection was not based on a unique optimum in the information criteria or entropy.

**Figure 2:**
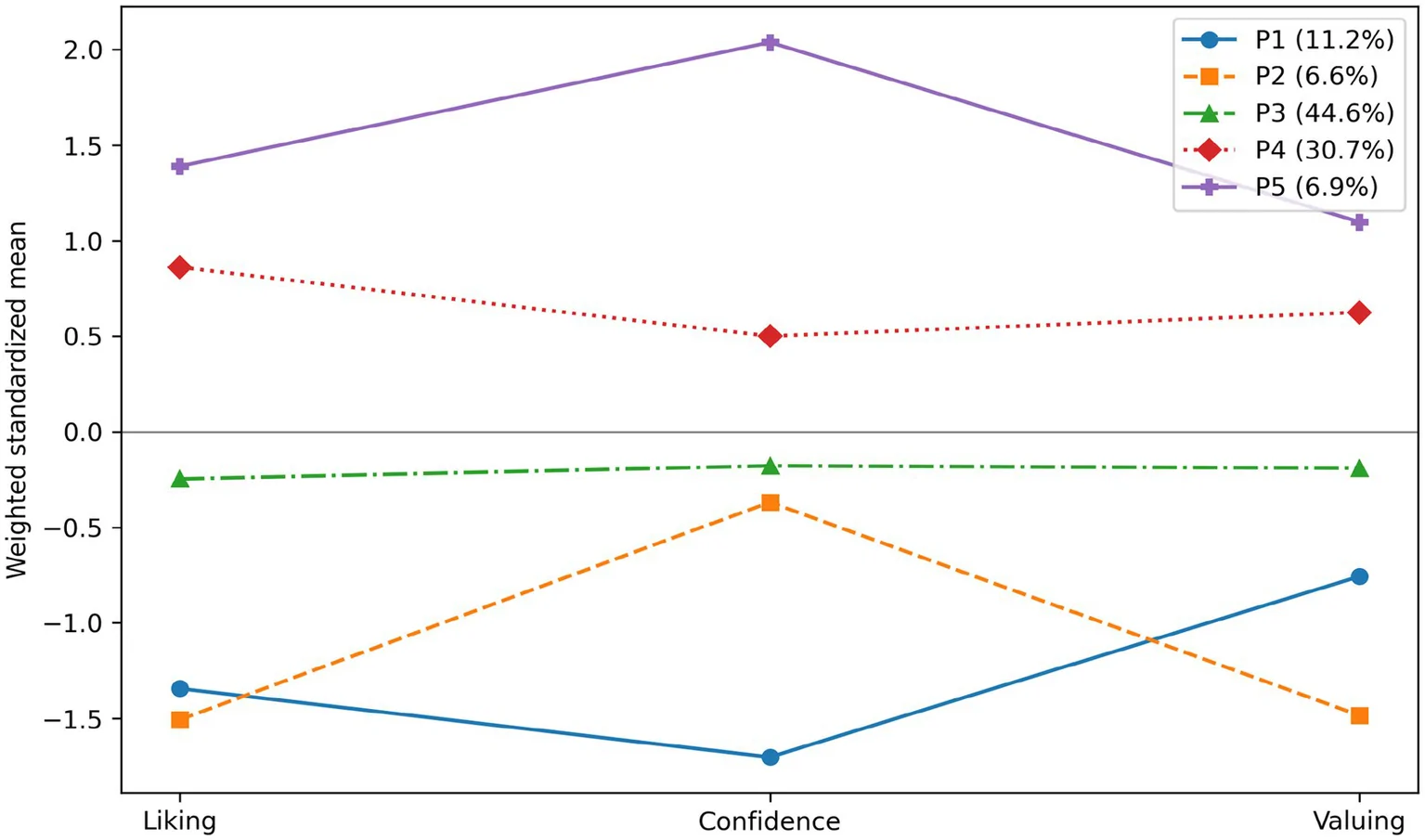
Weighted standardized means for the retained five-profile solution. Profiles 3, 4, and 5 differ mainly in level, whereas Profiles 1 and 2 cross: Profile 2 is higher on confidence and lower on valuing than Profile 1, indicating configurational rather than purely ordinal heterogeneity at the low end of the distribution. The legend reports model-estimated weighted prevalence; Table 2 separately reports modal weighted percentages.

Two sets of sensitivity analyses evaluated dependence on the measurement specification. First, enumeration was repeated across four covariance structures for one through eight profiles: equal diagonal (EEI; the retained specification), profile-specific diagonal (VVI), equal unrestricted (EEE), and profile-specific unrestricted (VVV). Each of the 32 models was estimated with 25 random starts after adding a diagonal ridge equal to 2% of the smallest indicator variance to every covariance matrix. An additive ridge was used because a coordinate-wise variance bound does not by itself protect the smallest eigenvalue of an unrestricted covariance matrix. These sensitivity values are internally comparable within the 2% grid but are not directly comparable with Table 1, whose primary models use only negligible numerical stabilization (1e-6). Regularization sensitivity was assessed by refitting VVI and VVV models with 80 random starts after increasing the additive ridge to 5% of the smallest indicator variance. Second, an auxiliary unweighted complete-case R/mclust fit was retained only as a software check of the equal-diagonal measurement specification; final reported inference comes from the authoritative Python analysis.

Information criteria decreased monotonically through eight profiles without a clear elbow, so AIC, BIC, and aBIC alone did not identify a stopping point. Model selection therefore relied on the joint pattern of profile size, classification quality, parsimony, the proximity of the best and second-best likelihood solutions, substantive distinctiveness, and sensitivity to alternative covariance structures. The information criteria were calculated from the weighted likelihood and were used as comparative model-selection indices rather than as design-adjusted hypothesis tests. Continuing improvement in information criteria was interpreted cautiously because finite normal mixture models can overextract classes when distributional assumptions are imperfect (Bauer and Curran, 2003).

TIMSS sampling weights and variance-estimation variables were incorporated using the official complex-sample information. Likelihood estimation used HOUWGT, the TIMSS sample-size-scaled transformation of the total student weight (TOTWGT). Because HOUWGT is a constant within-country rescaling of TOTWGT, it preserves relative weights and weighted point estimates while keeping the likelihood on the sample-size scale (Fishbein et al., 2025). This was verified in the analysis file: WGTFAC3 was 1.00 for every student and the HOUWGT-to-TOTWGT ratio was constant to within 7.2e-09. Design-based uncertainty used the official Full JK2 variables JKZONE and JKREP. The 71 variance strata generated 142 complementary replicate weights. For every replicate, the five-profile measurement model, classification-error matrix, main three-step model, and belonging-excluded three-step model were re-estimated; replicate classes were aligned to the full-sample solution before variance estimation.

To address RQ2, school belonging, bullying victimization, gender, and home educational resources were entered in a maximum-likelihood classification-error-corrected three-step multinomial model in the sense of Vermunt (2010), with the bias-adjusted framework further described by Bakk et al. (2013). Continuous covariates were standardized once in the full three-step sample and held fixed across replicates; odds ratios therefore represent a one-standard-deviation difference, and gender contrasts boys with girls. Students missing any covariate were excluded (*N* = 4,739). The full measurement and structural models were re-estimated for all 142 complementary Full JK2 replicates. Predictor-level omnibus inference used the finite-design-degrees-of-freedom-adjusted Wald statistic F__adj_ = W(d - q + 1)/(dq), referred to F(q, d - q + 1). With d = 71 design degrees of freedom and q = 4 profile-membership restrictions, all omnibus tests use *F*(4, 68). Pairwise contrasts were examined only after the omnibus test and Benjamini-Hochberg false-discovery-rate adjustment was applied separately to the 10 contrasts within each predictor family (Benjamini and Hochberg, 1995).

## 4 Results

### 4.1 Profile enumeration and interpretation

Fit statistics for the one- through eight-profile equal-variance models are presented in Table 1 and Figure 1. AIC, BIC, and aBIC decreased monotonically and did not show a clear elbow. The five-profile model had log-likelihood = −28,431.18, AIC = 56,906.37, BIC = 57,048.94, aBIC = 56,979.03, and entropy = 0.787. Information criteria were therefore not treated as identifying a unique stopping point.

The five-profile solution was retained after weighing fit against profile size, classification quality, parsimony, substantive distinctiveness, and sensitivity to covariance assumptions. Relative to four profiles, BIC decreased by 260.13 points and entropy increased from 0.769 to 0.787, while the smallest modal weighted profile was 5.64%. The five-to-six-profile BIC improvement remained nontrivial (206.33 points), so the decision was not based on a statistical elbow. However, the six-profile solution lowered entropy to 0.753 and produced a modal weighted profile of 4.39%. The complete weighted five-versus-six cross-classification (Supplementary Table S4) showed that the central Slightly Below-Average profile divided 54.2%/45.8% and the Positive profile divided 44.1%/55.9% between adjacent six-profile classes; the peripheral profiles were predominantly retained rather than forming a clearly new configuration. Seven- and eight-profile solutions fragmented the sample further, with modal weighted profiles below 1%. The five-profile solution was therefore retained as a parsimonious descriptive classification, not as a uniquely recovered population typology.

Sensitivity to the covariance specification. Supplementary Table S2 and Supplementary Figure S1 report the complete 32-model grid. The lowest BIC in the 2% additive-ridge grid was 54,494.27 for the VVV 8-profile model, 2,585.98 points below the EEI five-profile grid value (57,080.25). The corresponding entropy was 0.841 and its smallest modal weighted class was 0.87%. Among the 16 grid cells with five or more profiles, 3 retained a smallest modal weighted class of at least 5%. Flexible covariance models therefore provided materially better density fit, but often did so with small classes and solutions that varied across starts or ridge settings. Classification quality does not override better distributional fit. The retained EEI model instead serves the study’s stated parsimonious descriptive-classification objective under a transparent covariance constraint.

Regularization sensitivity confirmed that the magnitude of the class-specific covariance advantage depended on the ridge setting. Increasing the additive ridge from 2 to 5% changed BIC by 605.08 points for VVI with five profiles and by 99.10 and 1,209.07 points for VVV with five and six profiles, respectively. In the 5% VVV fits, the proportions of starts ending at or near the ridge scale were 13.8, 57.5, 88.8, 98.8, and 100.0% for four through eight profiles. Thus, alternative covariance structures cannot be dismissed, but their enumeration and class boundaries are regularization-sensitive. The retained model is presented alongside, rather than as a replacement for, that distributional-fit evidence.

Auxiliary software check. An unweighted complete-case R/mclust fit (*N* = 4,713; 60 random starts) returned five equal-diagonal profile proportions of 6.4, 6.6, 12.4, 29.4, and 45.3%. These were similar in rank and scale to the final weighted modal proportions (5.6, 6.6, 11.2, 30.4, 46.2%). The default single hierarchical start converged to a local maximum 52.22 log-likelihood units below the best random-start solution, underscoring the importance of multiple starts. This auxiliary check was not used to calculate the final Full JK2 inference.

The retained model was re-estimated with 1,000 random starts. The absolute difference between its best and second-best log-likelihood values was 0.00004. Overall entropy was 0.787. Average posterior probabilities ranged from 0.796 to 0.884; modal weighted percentages ranged from 5.64 to 46.20%, and model-estimated weighted prevalence ranged from 6.57 to 44.62% (Table 2). Profile 2 had the lowest AvePP (0.796) and classification-error correct-assignment probability (0.684), so contrasts involving this profile are interpreted cautiously. Figure 2 displays the weighted standardized indicator means and model-estimated weighted prevalence.

**Table 2:** Characteristics and classification quality of the retained five-profile solution.

Profile	Descriptive label	n	Unwt. %	Modal wt. %	AvePP	SLM z	SCM z	SVM z	Estimated wt. %
P1	Low overall/very low confidence	590	12.2	11.2	0.863	−1.34	−1.70	−0.76	11.24
P2	Low liking-value/relatively higher confidence	308	6.4	5.6	0.796	−1.51	−0.37	−1.49	6.57
P3	Slightly below-average	2,240	46.5	46.2	0.864	−0.25	−0.18	−0.19	44.62
P4	Positive	1,381	28.7	30.4	0.854	0.86	0.50	0.63	30.67
P5	Highly positive	301	6.2	6.6	0.884	1.39	2.04	1.10	6.91

AvePP, average posterior probability; SLM, Students Like Learning Mathematics; SCM, Students Confident in Mathematics; SVM, Students Value Mathematics. Overall weighted entropy = 0.787. Modal weighted % is based on modal class assignment. Estimated weighted % is the model-estimated latent-profile prevalence and corresponds to Figure 2. Profile labels are descriptive and do not imply fixed types or adaptive functioning.

Profile 1, labeled Low Overall / Very Low Confidence, had a model-estimated weighted prevalence of 11.24%. All three attitude scores were below the sample mean, with the largest deviation for confidence (SLM z = −1.34, SCM z = −1.70, SVM z = −0.76). The label is descriptive of the observed scale pattern and does not imply an adaptive or maladaptive outcome.

Profile 2, labeled Low Liking-Value / Relatively Higher Confidence, had a model-estimated weighted prevalence of 6.57%. Liking and valuing were both very low (z = −1.51 and −1.49), whereas confidence, although below the sample mean, was comparatively higher (z = −0.37). This profile therefore differed from Profile 1 in the relative alignment of the three attitude dimensions.

Profile 3, labeled Slightly Below-Average, was the largest group (model-estimated weighted prevalence = 44.62%). Scores on liking, confidence, and valuing were all modestly below the sample mean and closely aligned (z = −0.25, −0.18, and −0.19, respectively).

Profile 4, labeled Positive, had a model-estimated weighted prevalence of 30.67% and showed above-average scores across liking (z = 0.86), confidence (z = 0.50), and valuing (z = 0.63).

Profile 5, labeled Highly Positive, had a model-estimated weighted prevalence of 6.91% and had the highest scores on all three indicators, particularly confidence (SLM z = 1.39, SCM z = 2.04, SVM z = 1.10).

Taken together, the five-profile solution contained three broadly ordered profiles (Slightly Below-Average, Positive, and Highly Positive) and two lower-attitude profiles with different within-profile configurations. Thus, the final solution reflects both level differences and a limited degree of configurational heterogeneity rather than five sharply distinct motivational types. Weighted descriptive statistics, missingness, and correlations among the continuous study variables are presented in Table 3.

**Table 3:** Weighted descriptive statistics, missingness, and correlations among continuous study variables.

Panel A. Descriptive statistics and missingness						
Var.	Construct	Valid N	Missing %	M	SD	Observed range
1	Liking mathematics	4,798	2.58	10.22	1.97	6.33–13.61
2	Confidence in mathematics	4,758	3.39	9.65	2.34	4.42–15.59
3	Valuing mathematics	4,773	3.09	9.40	2.10	3.61–12.94
4	School belonging	4,767	3.21	10.09	2.02	4.36–13.94
5	Student bullying (official direction)	4,808	2.38	9.45	1.65	3.25–12.89
6	Home educational resources	4,833	1.87	10.23	1.66	4.30–13.53

Panel B. Weighted correlations						
Var.	1	2	3	4	5	6
1	1.000					
2	0.670	1.000				
3	0.608	0.435	1.000			
4	0.465	0.277	0.391	1.000		
5	0.101	0.103	0.100	0.338	1.000	
6	0.082	0.221	0.108	−0.010	0.047	1.000

Variable numbering: 1 = Liking mathematics; 2 = Confidence in mathematics; 3 = Valuing mathematics; 4 = Perceived school belonging; 5 = Student bullying; 6 = Home educational resources. Statistics are weighted with the official TIMSS HOUWGT. BSBGSB is shown in its official TIMSS direction, in which higher scores indicate less frequent bullying victimization. For the three-step regression, the standardized bullying score was sign-reversed so that higher analytic scores represented more victimization.

### 4.2 Profile membership and student characteristics

The four student characteristics were examined with design-adjusted, classification-error-corrected three-step multinomial models. Perceived school belonging differentiated profile membership overall, *F*(4, 68) = 47.74, *p* < 0.001; home educational resources also showed an omnibus association, *F*(4, 68) = 15.61, *p* < 0.001. Gender showed a smaller overall association, *F*(4, 68) = 2.88, *p* = 0.029. Bullying victimization did not significantly differentiate profiles after adjustment for the other covariates, *F*(4, 68) = 1.78, *p* = 0.143 (Table 4).

**Table 4:** Design-adjusted omnibus tests for correlates of profile membership.

Predictor	Adjusted Wald F	df1	df2	*p*
Perceived school belonging	47.74	4	68	< 0.001
Bullying victimization	1.78	4	68	0.143
Gender (boy vs. girl)	2.88	4	68	0.029
Home educational resources	15.61	4	68	< 0.001

Omnibus inference uses the finite-design-degrees-of-freedom-adjusted Full JK2 Wald F statistic. With d = 71 design degrees of freedom and q = 4 restrictions, F__adj_ = W(d - q + 1)/(dq) and is referred to F(4, 68). Continuous predictors were standardized once and held fixed across replicates. Pairwise contrasts were examined only after the omnibus tests and adjusted separately within each 10-contrast predictor family using the Benjamini-Hochberg false discovery rate. In the model excluding perceived school belonging, bullying victimization yielded F(4, 68) = 6.02, *p* < 0.001.

Perceived school belonging was the most consistent correlate: all 10 pairwise contrasts survived BH-FDR correction, although the Highly Positive-versus-Positive contrast was marginal (OR = 1.36, 95% CI [1.00, 1.84], p__FDR_ = 0.048). Higher belonging was generally associated with greater odds of membership in the Slightly Below-Average, Positive, or Highly Positive profiles rather than the lower-attitude profiles. A one-standard-deviation increase in belonging was associated with 6.04 times higher odds of Highly Positive rather than Low Overall / Very Low Confidence membership (95% CI [4.19, 8.72]) and 9.39 times higher odds of Positive rather than Low Liking-Value / Relatively Higher Confidence membership (95% CI [6.27, 14.06]).

The comparison between the two lowest profiles was nonmonotonic: higher belonging was associated with lower odds of Low Liking-Value / Relatively Higher Confidence rather than Low Overall / Very Low Confidence membership (OR = 0.47, 95% CI [0.32, 0.69], p__FDR_ < 0.001). Because Profile 2 was the least cleanly separated profile (AvePP = 0.796; correct-assignment probability = 0.684), this contrast should be treated as provisional rather than as evidence of a distinct causal or developmental process.

Home educational resources primarily differentiated the two upper profiles from the lower and near-average profiles. Seven of 10 pairwise contrasts survived BH-FDR adjustment. A one-standard-deviation increase was associated with higher odds of Highly Positive rather than Slightly Below-Average membership (OR = 2.55, 95% CI [2.01, 3.24]) and Positive rather than Slightly Below-Average membership (OR = 1.46, 95% CI [1.14, 1.87]). Highly Positive also differed from Positive (OR = 1.75, 95% CI [1.32, 2.32]).

The gender omnibus test was statistically significant, but only one of 10 pairwise contrasts survived BH-FDR correction: boys had higher odds than girls of Positive rather than Low Overall / Very Low Confidence membership (OR = 1.78, 95% CI [1.22, 2.60], pp__FDR_ = 0.035). No bullying-victimization contrast survived FDR adjustment in the simultaneous model. When the model was re-estimated without perceived school belonging under a separate 142-replicate Full JK2 run, bullying victimization did differentiate profiles, *F*(4, 68) = 6.02, *p* < 0.001. On the official BSBGSB metric, for which higher values indicate less frequent victimization, the weighted correlation with perceived school belonging was r = 0.338. After sign reversal so that higher analytic scores represented greater victimization, the same correlation was r = −0.338. This specification-dependent conditional association does not establish mediation, confounding, or temporal ordering.

## 5 Discussion

This study re-examined heterogeneity in mathematics attitudes among Turkish eighth-grade students using the three official TIMSS attitude scale scores and the TIMSS complex-sample design. The revised analysis identified five profiles. Three profiles were broadly ordered by overall attitude level, while two lower-attitude profiles differed in the relative position of confidence versus liking and value. Perceived school belonging was the most consistent correlate of profile membership, home educational resources differentiated the upper profiles from lower profiles, gender showed limited profile-specific differences, and bullying victimization did not show a statistically significant omnibus association in the simultaneous model.

### 5.1 The retained five-profile classification: predominantly level differences with two low-attitude configurations

The retained solution is more cautious than the original six-profile interpretation. The Slightly Below-Average, Positive, and Highly Positive profiles were largely parallel across liking, confidence, and valuing, indicating quantitative differences in the overall positivity of mathematics attitudes. This pattern suggests that much of the heterogeneity in the sample is level-based rather than composed of sharply different motivational types. At the same time, the two lowest profiles crossed: Profile 1 showed especially low confidence with somewhat less negative valuing, whereas Profile 2 showed very low liking and valuing but relatively higher confidence. These two configurations support retaining a person-centered perspective while avoiding the claim that every profile represents a qualitatively distinct motivational orientation.

EVT provides a useful framework for interpreting the two low-attitude configurations because expectancy-related confidence and value-related appraisals need not align (Wigfield and Eccles, 2000; Eccles and Wigfield, 2020). However, the revised profile labels deliberately remain descriptive. In particular, the data do not directly measure an ‘instrumental motivational orientation,’ and the former Instrumentally Motivated label has been removed. Similarly, no profile is described as adaptive or maladaptive because the study does not include external achievement, persistence, or well-being criteria.

The Turkish high-stakes context remains relevant as background rather than as an explanation established by the data. Mathematics is consequential for secondary-school placement, which may heighten perceived importance, but the present cross-sectional analysis cannot determine whether examination pressure causes the observed configurations. Future work that directly measures examination pressure, cost, utility value, and longitudinal change would be needed to test that mechanism.

### 5.2 School belonging as the most consistent correlate

Perceived school belonging differentiated all 10 profile pairs after FDR adjustment, although the Highly Positive-versus-Positive contrast was marginal (p_FDR = 0.048). The association generally favored membership in the slightly below-average, positive, or highly positive profiles over the two lower-attitude profiles. The comparison between the two low profiles was nonmonotonic: higher belonging favored the Low Overall / Very Low Confidence profile over the Low Liking-Value / Relatively Higher Confidence profile. This pattern and Profile 2’s lower classification precision reinforce the need to interpret belonging as profile-specific rather than as a simple linear marker of attitude positivity.

SDT offers a cautious interpretive lens for this pattern because experiences of acceptance and connection are conceptually related to relatedness (Ryan and Deci, 2000). Nevertheless, the TIMSS school-belonging scale is an individual-level perceptual measure and is not a direct measure of SDT need satisfaction. The results therefore support describing belonging as the most consistent correlate of profile membership, not as a causal school-climate effect or a direct test of SDT.

### 5.3 Bullying victimization after simultaneous adjustment for school belonging

After correcting the direction of the official TIMSS bullying scale, standardizing it so that higher analytic scores represented more victimization, incorporating the complex sample design, and controlling the false discovery rate, bullying victimization did not significantly differentiate profile membership in the simultaneous model. When perceived school belonging was omitted, bullying victimization did differentiate profiles, *F*(4, 68) = 6.02, *p* < 0.001. Because the regression-oriented victimization score is negatively correlated with perceived school belonging (r = −0.338; equivalently, the official less-victimization BSBGSB score correlates positively with belonging at r = 0.338), the change shows sensitivity to simultaneous adjustment for belonging. It does not establish mediation, confounding, causal direction, or a single underlying ‘true’ bullying effect. Longitudinal or experimental evidence would be required to evaluate temporal or indirect mechanisms.

### 5.4 Gender and home educational resources

Gender showed a statistically significant omnibus association but little evidence of widespread pairwise differentiation. Only the Positive versus Low Overall / Very Low Confidence contrast survived FDR adjustment, with boys showing higher odds of membership in the Positive profile. This isolated result is consistent with the broader literature on gender differences in mathematics confidence (Mejía-Rodríguez et al., 2021; Rodríguez et al., 2020) but does not justify a general claim that one gender is more likely to occupy more positive profiles.

Home educational resources showed a clearer gradient. Profiles 1, 2, and 3 did not differ reliably from one another on this covariate, whereas greater resources were associated with higher odds of membership in the Positive and Highly Positive profiles. The largest effects involved the Highly Positive profile. These associations are consistent with research linking educational resources and socioeconomic conditions with mathematics outcomes (Broer et al., 2019; Niehues et al., 2020), but the cross-sectional design does not identify the mechanisms responsible for the pattern.

### 5.5 Implications for practice and policy

The revised profiles suggest that students with broadly low mathematics attitudes are not homogeneous. One lower-attitude group was distinguished by especially low confidence, whereas another combined very low liking and valuing with comparatively higher confidence. These patterns may help generate hypotheses about different forms of support, but the present study did not evaluate interventions or external outcomes. Accordingly, profile-specific instructional implications should be treated as hypotheses for future testing rather than prescriptions derived from causal evidence.

The association with perceived school belonging may motivate attention to supportive and inclusive classroom relationships, but the analysis does not estimate a school-level climate effect. BSBGSSB is an individual student’s perception, and students were not aggregated into a school-level construct. Similarly, the home-resource findings point to socioeconomic patterning without showing that any particular school intervention would change profile membership. Multilevel and intervention studies are needed before drawing school-level policy conclusions.

### 5.6 Limitations and future directions

Several limitations qualify the findings. First, the study is cross-sectional and cannot establish causal direction. Second, all focal constructs are based on student self-report. Third, and most consequentially, the five-profile solution depends on the three official TIMSS attitude scales and on an equal-variance, zero-covariance mixture specification. More flexible covariance structures achieved markedly lower BIC in the 2% additive-ridge grid, while often producing small classes and solutions sensitive to random starts or the 5% ridge condition. The retained model is therefore a parsimonious descriptive classification under stated constraints, not the best-fitting representation of the joint density or a uniquely recovered population structure. Fourth, Profile 2 was the least cleanly separated profile (AvePP = 0.796; correct-assignment probability = 0.684), so findings involving it should be considered provisional. Fifth, the three-step stage used complete covariate information (N = 4,739). Sixth, no achievement, persistence, or well-being outcomes were included, so the labels do not denote adaptive functioning. Seventh, perceived school belonging is an individual-level measure, not school climate. Finally, the Türkiye sample and high-stakes context may limit cross-national generalizability.

Future research could examine profile stability with longitudinal or latent-transition models, link the profiles to achievement and later STEM choices, and test multilevel mixture models that separate individual belonging from school-level context. More differentiated bullying measures could distinguish verbal, relational, physical, and cyber victimization. Formal longitudinal mediation models could also test whether changes in belonging help explain associations between bullying experiences and later mathematics attitudes. Such analyses would address mechanisms that the present concurrent three-step model cannot identify.

## 6 Conclusion

Mathematics attitudes among Turkish eighth-grade students were represented by five profiles in the revised analysis: Low Overall / Very Low Confidence, Low Liking-Value / Relatively Higher Confidence, Slightly Below-Average, Positive, and Highly Positive. Three profiles primarily reflected ordered differences in overall attitude level, whereas the two lowest profiles differed in the relative alignment of confidence, liking, and value. This combination of level and configuration differences supports a person-centered description while arguing against overly strong typological labels.

Perceived school belonging was the most consistent correlate of profile membership, and home educational resources differentiated the positive end of the profile distribution. Gender showed only limited profile-specific differentiation after multiplicity correction. Bullying victimization did not show a statistically significant omnibus association in the simultaneous model after its direction was corrected and the TIMSS complex sample design was incorporated. These are conditional cross-sectional associations and should not be interpreted as causal pathways.

The revised findings suggest that mathematics attitudes are heterogeneous but that this heterogeneity is more parsimoniously described than in the original item-level analysis. Using official TIMSS scale scores, design-based variance estimation, classification-error correction, and multiplicity control yields a more defensible account of the retained profile classification and its student-level correlates.

## Data Availability

The TIMSS 2023 Türkiye public-use data are available from the IEA/TIMSS 2023 International Database and are not redistributed here. The supplementary code-and-output archive contains the authoritative Python analysis script, a self-contained Google Colab runner, locked package requirements, algorithm-verification tests, the run manifest with code and data SHA-256 hashes, the completed analysis audit, profile assignments and posterior probabilities, all 142 JK2 coefficient replicates and covariance matrices, omnibus and BH-FDR results, the complete covariance-sensitivity grids and start diagnostics, full five-versus-six-profile cross-classifications, and the figures. Running the documented command on the public-use SAV file regenerates the deposited numerical outputs. All analysis materials used for the reported results are supplied in the supplementary archive.
