# Supplementary material for: Latent mathematics attitude profiles among eighth-grade students: associations with school belonging and bullying
Source: Front Psychol. 2026 Sep 24;17:1938974. doi: 10.3389/fpsyg.2026.1938974 (PMC13646153; doi:10.3389/fpsyg.2026.1938974)
Supplement: Supplementary file 1 [file Data_Sheet_1.zip › Data Sheet 1/Frontiers_1938974_SUPPLEMENTARY_TABLES_AND_FIGURES.docx]

Supplementary Tables and Figures

**Manuscript 1938974**

# Scope and provenance

Every number and figure in this supplement was generated by the same authoritative full analysis (code SHA-256 cf096f67554a364ac5c37a45181c6ddf75034e4d0c3427f0a700e5abf7b0cee6; data SHA-256 0a74614d400eeb278156892c2a88bd78e223fecf76981527175a191cc164f20f). The public-use TIMSS SAV file is not redistributed. The accompanying archive contains the exact machine-readable tables, start-level diagnostics, analysis audit, and run manifest.

Primary enumeration used the equal-diagonal model with negligible numerical stabilization (1e-6). The covariance grid adds a diagonal ridge equal to 2% of the smallest indicator variance to every covariance matrix; Table S3 repeats the class-specific structures at 5%. The sensitivity results describe alternative representations of the joint density and are not overridden by entropy or class-size considerations.

# Figure S1. Covariance-structure sensitivity

Note. BIC trajectories for EEI (equal diagonal), VVI (profile-specific diagonal), EEE (equal unrestricted), and VVV (profile-specific unrestricted) models estimated with the 2% additive diagonal ridge. Values are reproduced in Table S2.

# Table S2. Complete covariance-structure grid (2% additive diagonal ridge)

## Panel A. Equal diagonal (EEI)

| **k** | **Par.** | **LogLik** | **AIC** | **BIC** | **aBIC** | **Ent.** | **Min n** | **Min modal %** | **Min est. %** | **Conv./starts** | **Near ridge** |
| --- | --- | --- | --- | --- | --- | --- | --- | --- | --- | --- | --- |
| 1 | 6 | -31202.61 | 62417.22 | 62456.10 | 62437.03 | - | 4820 | 100.00 | 100.00 | 25/25 | 0 |
| 2 | 10 | -29531.78 | 59083.55 | 59148.36 | 59116.58 | 0.693 | 2371 | 48.51 | 48.45 | 25/25 | 0 |
| 3 | 14 | -28828.98 | 57685.96 | 57776.69 | 57732.20 | 0.751 | 1053 | 20.00 | 20.76 | 25/25 | 0 |
| 4 | 18 | -28632.93 | 57301.86 | 57418.51 | 57361.31 | 0.743 | 276 | 6.02 | 6.68 | 25/25 | 0 |
| 5 | 22 | -28446.84 | 56937.67 | 57080.25 | 57010.34 | 0.773 | 281 | 5.17 | 6.26 | 25/25 | 0 |
| 6 | 26 | -28341.37 | 56734.73 | 56903.23 | 56820.61 | 0.720 | 168 | 2.88 | 3.13 | 25/25 | 0 |
| 7 | 30 | -28257.33 | 56574.66 | 56769.07 | 56673.74 | 0.757 | 27 | 0.59 | 0.57 | 25/25 | 0 |
| 8 | 34 | -28212.29 | 56492.58 | 56712.92 | 56604.88 | 0.733 | 29 | 0.61 | 0.59 | 25/25 | 0 |

Note. N = 4,820; 25 random starts per cell. Min modal % is the HOUWGT-weighted percentage in the smallest modal class; Min est. % is the smallest model-estimated latent prevalence. Near ridge counts starts whose minimum covariance eigenvalue ended within 5% of the added ridge scale. Complete precision and errors, if any, are retained in the CSV output.

## Panel B. Profile-specific diagonal (VVI)

| **k** | **Par.** | **LogLik** | **AIC** | **BIC** | **aBIC** | **Ent.** | **Min n** | **Min modal %** | **Min est. %** | **Conv./starts** | **Near ridge** |
| --- | --- | --- | --- | --- | --- | --- | --- | --- | --- | --- | --- |
| 1 | 6 | -31202.61 | 62417.22 | 62456.10 | 62437.03 | - | 4820 | 100.00 | 100.00 | 25/25 | 0 |
| 2 | 13 | -29490.63 | 59007.27 | 59091.51 | 59050.20 | 0.700 | 2103 | 45.89 | 45.64 | 25/25 | 0 |
| 3 | 20 | -28513.95 | 57067.91 | 57197.52 | 57133.96 | 0.749 | 1135 | 24.03 | 25.34 | 25/25 | 0 |
| 4 | 27 | -28185.66 | 56425.33 | 56600.30 | 56514.50 | 0.772 | 306 | 5.45 | 5.29 | 25/25 | 1 |
| 5 | 34 | -27892.92 | 55853.83 | 56074.17 | 55966.13 | 0.705 | 394 | 8.58 | 8.25 | 25/25 | 12 |
| 6 | 41 | -27594.52 | 55271.05 | 55536.75 | 55406.46 | 0.737 | 305 | 5.42 | 5.28 | 25/25 | 19 |
| 7 | 48 | -27473.06 | 55042.12 | 55353.18 | 55200.66 | 0.751 | 71 | 1.40 | 2.44 | 25/25 | 25 |
| 8 | 55 | -27285.19 | 54680.39 | 55036.82 | 54862.05 | 0.775 | 76 | 1.47 | 2.61 | 25/25 | 25 |

Note. N = 4,820; 25 random starts per cell. Min modal % is the HOUWGT-weighted percentage in the smallest modal class; Min est. % is the smallest model-estimated latent prevalence. Near ridge counts starts whose minimum covariance eigenvalue ended within 5% of the added ridge scale. Complete precision and errors, if any, are retained in the CSV output.

## Panel C. Equal unrestricted (EEE)

| **k** | **Par.** | **LogLik** | **AIC** | **BIC** | **aBIC** | **Ent.** | **Min n** | **Min modal %** | **Min est. %** | **Conv./starts** | **Near ridge** |
| --- | --- | --- | --- | --- | --- | --- | --- | --- | --- | --- | --- |
| 1 | 9 | -28692.99 | 57403.97 | 57462.30 | 57433.70 | - | 4820 | 100.00 | 100.00 | 25/25 | 0 |
| 2 | 13 | -28599.55 | 57225.09 | 57309.34 | 57268.03 | 0.928 | 143 | 2.51 | 3.28 | 25/25 | 0 |
| 3 | 17 | -28387.22 | 56808.44 | 56918.61 | 56864.59 | 0.859 | 294 | 6.14 | 7.19 | 25/25 | 0 |
| 4 | 21 | -28319.54 | 56681.09 | 56817.18 | 56750.45 | 0.861 | 125 | 2.21 | 2.57 | 25/25 | 0 |
| 5 | 25 | -28264.51 | 56579.02 | 56741.03 | 56661.59 | 0.679 | 111 | 1.88 | 2.14 | 25/25 | 0 |
| 6 | 29 | -28208.28 | 56474.55 | 56662.49 | 56570.34 | 0.689 | 123 | 2.11 | 2.35 | 25/25 | 0 |
| 7 | 33 | -28184.47 | 56434.94 | 56648.80 | 56543.94 | 0.692 | 34 | 0.73 | 1.02 | 25/25 | 0 |
| 8 | 37 | -28115.25 | 56304.50 | 56544.28 | 56426.71 | 0.720 | 17 | 0.39 | 0.47 | 25/25 | 0 |

Note. N = 4,820; 25 random starts per cell. Min modal % is the HOUWGT-weighted percentage in the smallest modal class; Min est. % is the smallest model-estimated latent prevalence. Near ridge counts starts whose minimum covariance eigenvalue ended within 5% of the added ridge scale. Complete precision and errors, if any, are retained in the CSV output.

## Panel D. Profile-specific unrestricted (VVV)

| **k** | **Par.** | **LogLik** | **AIC** | **BIC** | **aBIC** | **Ent.** | **Min n** | **Min modal %** | **Min est. %** | **Conv./starts** | **Near ridge** |
| --- | --- | --- | --- | --- | --- | --- | --- | --- | --- | --- | --- |
| 1 | 9 | -28692.99 | 57403.97 | 57462.30 | 57433.70 | - | 4820 | 100.00 | 100.00 | 25/25 | 0 |
| 2 | 19 | -28423.01 | 56884.02 | 57007.15 | 56946.78 | 0.298 | 1414 | 27.66 | 39.96 | 25/25 | 0 |
| 3 | 29 | -28047.40 | 56152.80 | 56340.73 | 56248.58 | 0.784 | 306 | 5.45 | 5.46 | 25/25 | 2 |
| 4 | 39 | -27744.17 | 55566.33 | 55819.07 | 55695.15 | 0.715 | 317 | 6.83 | 10.31 | 25/25 | 8 |
| 5 | 49 | -27642.04 | 55382.07 | 55699.62 | 55543.92 | 0.771 | 79 | 1.51 | 2.28 | 25/25 | 24 |
| 6 | 59 | -27036.55 | 54191.11 | 54573.46 | 54385.98 | 0.958 | 120 | 2.11 | 1.91 | 25/25 | 25 |
| 7 | 69 | -26967.34 | 54072.67 | 54519.83 | 54300.57 | 0.940 | 39 | 0.81 | 1.69 | 25/25 | 25 |
| 8 | 79 | -26912.15 | 53982.31 | 54494.27 | 54243.24 | 0.841 | 42 | 0.87 | 1.74 | 25/25 | 25 |

Note. N = 4,820; 25 random starts per cell. Min modal % is the HOUWGT-weighted percentage in the smallest modal class; Min est. % is the smallest model-estimated latent prevalence. Near ridge counts starts whose minimum covariance eigenvalue ended within 5% of the added ridge scale. Complete precision and errors, if any, are retained in the CSV output.

# Table S3. Regularization sensitivity (5% additive diagonal ridge)

## Panel A. Profile-specific diagonal (VVI)

| **k** | **Par.** | **LogLik** | **AIC** | **BIC** | **aBIC** | **Ent.** | **Min n** | **Min modal %** | **Min est. %** | **Conv./starts** | **Near ridge** |
| --- | --- | --- | --- | --- | --- | --- | --- | --- | --- | --- | --- |
| 1 | 6 | -31207.97 | 62427.95 | 62466.83 | 62447.76 | - | 4820 | 100.00 | 100.00 | 80/80 | 0 |
| 2 | 13 | -29512.72 | 59051.43 | 59135.68 | 59094.37 | 0.684 | 2049 | 44.68 | 44.36 | 80/80 | 0 |
| 3 | 20 | -28580.16 | 57200.33 | 57329.94 | 57266.38 | 0.730 | 1135 | 22.26 | 23.47 | 80/80 | 0 |
| 4 | 27 | -28378.46 | 56810.92 | 56985.89 | 56900.10 | 0.653 | 574 | 12.46 | 13.34 | 80/80 | 0 |
| 5 | 34 | -28195.46 | 56458.92 | 56679.25 | 56571.21 | 0.690 | 306 | 5.45 | 5.07 | 80/80 | 2 |
| 6 | 41 | -28068.39 | 56218.78 | 56484.48 | 56354.20 | 0.707 | 130 | 2.28 | 2.25 | 80/80 | 6 |
| 7 | 48 | -27905.71 | 55907.43 | 56218.49 | 56065.97 | 0.742 | 134 | 2.36 | 2.38 | 80/80 | 32 |
| 8 | 55 | -27796.25 | 55702.50 | 56058.93 | 55884.16 | 0.768 | 39 | 0.88 | 0.99 | 80/80 | 61 |

Note. N = 4,820; 80 random starts per cell. Definitions match Table S2. These fits diagnose sensitivity to the regularization scale; they are not a second primary enumeration.

## Panel B. Profile-specific unrestricted (VVV)

| **k** | **Par.** | **LogLik** | **AIC** | **BIC** | **aBIC** | **Ent.** | **Min n** | **Min modal %** | **Min est. %** | **Conv./starts** | **Near ridge** |
| --- | --- | --- | --- | --- | --- | --- | --- | --- | --- | --- | --- |
| 1 | 9 | -28719.16 | 57456.32 | 57514.65 | 57486.05 | - | 4820 | 100.00 | 100.00 | 80/80 | 0 |
| 2 | 19 | -28483.72 | 57005.45 | 57128.58 | 57068.20 | 0.985 | 160 | 2.80 | 2.63 | 80/80 | 0 |
| 3 | 29 | -28208.11 | 56474.21 | 56662.15 | 56569.99 | 0.773 | 306 | 5.45 | 5.37 | 80/80 | 1 |
| 4 | 39 | -28041.01 | 56160.02 | 56412.76 | 56288.83 | 0.788 | 125 | 2.35 | 4.45 | 80/80 | 11 |
| 5 | 49 | -27691.59 | 55481.17 | 55798.72 | 55643.01 | 0.940 | 129 | 2.25 | 2.29 | 80/80 | 46 |
| 6 | 59 | -27641.09 | 55400.18 | 55782.53 | 55595.05 | 0.932 | 20 | 0.41 | 0.96 | 80/80 | 71 |
| 7 | 69 | -27571.43 | 55280.86 | 55728.02 | 55508.76 | 0.907 | 22 | 0.47 | 1.03 | 80/80 | 79 |
| 8 | 79 | -27544.60 | 55247.21 | 55759.17 | 55508.14 | 0.920 | 12 | 0.26 | 0.34 | 80/80 | 80 |

Note. N = 4,820; 80 random starts per cell. Definitions match Table S2. These fits diagnose sensitivity to the regularization scale; they are not a second primary enumeration.

# Table S4. Full five-versus-six-profile cross-classification

## Panel A. Unweighted student counts

| **Five-profile** | **Six-profile P1** | **Six-profile P2** | **Six-profile P3** | **Six-profile P4** | **Six-profile P5** | **Six-profile P6** | **Row total** |
| --- | --- | --- | --- | --- | --- | --- | --- |
| P1 | 485 | 1 | 104 | 0 | 0 | 0 | 590 |
| P2 | 3 | 242 | 63 | 0 | 0 | 0 | 308 |
| P3 | 0 | 2 | 1231 | 1007 | 0 | 0 | 2240 |
| P4 | 0 | 0 | 0 | 616 | 765 | 0 | 1381 |
| P5 | 0 | 0 | 0 | 5 | 46 | 250 | 301 |

Note. Rows use the retained five-profile ordering; columns use the six-profile ordering by mean attitude level. N = 4,820.

## Panel B. HOUWGT-weighted row percentages

| **Five-profile** | **Six-profile P1** | **Six-profile P2** | **Six-profile P3** | **Six-profile P4** | **Six-profile P5** | **Six-profile P6** | **Row total** |
| --- | --- | --- | --- | --- | --- | --- | --- |
| P1 | 81.13 | 0.11 | 18.76 | 0.00 | 0.00 | 0.00 | 100.00 |
| P2 | 1.11 | 77.27 | 21.62 | 0.00 | 0.00 | 0.00 | 100.00 |
| P3 | 0.00 | 0.05 | 54.17 | 45.79 | 0.00 | 0.00 | 100.00 |
| P4 | 0.00 | 0.00 | 0.00 | 44.06 | 55.94 | 0.00 | 100.00 |
| P5 | 0.00 | 0.00 | 0.00 | 2.07 | 16.37 | 81.56 | 100.00 |

Note. Percentages sum to 100 within each five-profile row, apart from rounding. Machine-readable count, HOUWGT weight-sum, and weighted-row-percentage matrices are included in the code-and-output archive. The cross-classification shows subdivision of adjacent ordered groups rather than a single clearly novel sixth configuration.

# Supplementary reproducibility note

The authoritative Python script generates Tables S2-S4 and Figure S1 in the same run as the primary enumeration and both Full JK2 structural models. completion_status.json records 142 JK2 replicates and the requested sensitivity stages; Z_Analysis_Audit.csv records the pre-specified completion checks. The separate verify_algorithms.py script checks key implementation identities independently of the empirical estimates.
